# Beta-lactam antibiotics administration among adult inpatients with a beta-lactam allergy label: incidence, predictors, and outcomes

**DOI:** 10.1017/ash.2024.68

**Published:** 2024-04-30

**Authors:** Naama Epstein-Rigbi, Sharon Ziv, Marina Bulanenkova, Ruth Bouganim, Ruthy Tal-Jasper, Dror Marchaim

**Affiliations:** 1 The Institute of Allergy, Immunology and Pediatric Pulmonology, Shamir (Assaf Harofeh) Medical Center, Beer Yaacov, Israel; 2 Faculty of Medicine, Tel-Aviv University, Tel-Aviv, Israel; 3 Department of Geriatrics, Shmuel Harofeh Medical Center, Beer Yaacov, Israel; 4 Unit of Infection Control, Shamir (Assaf Harofeh) Medical Center, Beer Yaacov, Israel

## Abstract

**Background:**

A beta-lactam antibiotics (BLA) allergy label is common, resulting in disadvantageous outcomes due to the usage of second-line antimicrobial agents. Noncontrolled case-series analyses report low rates of hypersensitivity reactions, following intentional/non-intentional BLA challenges among labeled inpatients. The study aims were to explore predictors and outcomes associated with hypersensitivity reactions following BLA challenge among BLA-allergic labeled inpatients.

**Methods:**

Retrospective cohort study (2019–2020) of adult (≥18 years) inpatients (Shamir Medical Center, Israel), labeled as allergic to ≥1 BLA, who received ≥1 dose/s of BLA during their stay. Independent predictors to develop allergic reactions and the independent associations of allergic reactions with clinical outcomes were queried by logistic and Cox regressions.

**Results:**

Of 9,670 inpatients (14,088 hospitalizations), 3,570 (37%) were labeled as allergic to ≥1 BLA. Of those, 1,171 (33%) patients received ≥1 BLA. The majority were women (67%), and the mean age was 69.3 ± 19.4 years. Only 30 patients (2.6%) developed a reaction, all mild. Independent predictors to develop an allergic reaction were documented reactions in the past, atopic background, antihistamines administration prior to the BLA challenge, and high risk for cross-reactivity, based on the BLA side chains, between the labeled and the challenged agents. Reaction upon the BLA challenge was not independently associated with any worse outcome.

**Conclusions:**

Despite the commonality of allergy labeling, and the commonality of BLA administration to labeled inpatients, hypersensitivity reactions were mild and rare. Interventional stewardship strategies for active BLA de-labeling among low-risk patients should be promoted, to improve patients’ and institutional health and fiscal outcomes.

## Introduction

Beta-lactam antibiotics (BLA) are among the oldest and most commonly prescribed drugs worldwide.^1^ BLA are considered first-line agents in many infectious clinical syndromes,^2^ due to their bactericidal, effective, safe, and well-tolerated profile.^3^ Non-BLA antimicrobial agents are frequently less efficacious and more toxic, promote the acquisition and dissemination of epidemiologically significant resistance traits, and are related to poorer clinical outcomes.^4–8^ BLA are also the most common drug class associated with an allergy label, with a reported prevalence of up to 25% among inpatients.^1,9–11^ However, it has been consistently reported that over 90% of BLA allergy-labeled patients can tolerate a BLA safely upon re-exposure; thus unnecessary usage of second-line agents could potentially be avoided.^12^

While most allergy labels to BLA are given during early childhood,^13^ usually due to a benign maculopapular rash,^11^ they persist in the medical records, frequently for the rest of the patient’s life, without further verification.^4^ Septic patients in hospitals frequently have severe acute illness indices, and the process of active de-labeling is not perceived as a priority in the acute state, despite the potential benefit of receiving BLA-based therapeutic regimens, instead of second-line agents.^14^ Moreover, even if the patient experienced an uneventful challenge to BLA, frequently the patient or his family continue reporting the allergy history upon re-admission, and the de-labeling process is overlooked.^8^ Additional potential barriers to proactive BLA de-labeling implementation, despite the multiple potential benefits, are the lack of sufficient allergy experts in many inpatient services and lack of patients’ education pertaining to the medical consequences associated with carrying such labels.^15^

There are few validated models for risk stratification, which promote BLA de-labeling among low-risk patients, but none of them are widely used (eg, PEN-FAST,^16^ CATALYST^17^). Due to the detrimental effects of BLA allergy mislabeling, there is an obvious need to prioritize a formal, risk-stratified allergy assessment protocol as part of any antimicrobials’ stewardship program. Our study’s aims were to analyze the descriptive epidemiology of BLA allergy labeling among inpatients, the BLA-challenged allergic reaction rate, its severity, and the risk factors and outcomes associated with BLA challenge among labeled inpatients.

## Methods

A retrospective cohort study was executed at Shamir (Assaf Harofeh) Medical Center (SMC), central Israel, a university-affiliated 904-bed acute-care institution, for calendar years 2019–2020. The study was approved by the local ethical institutional review board prior to its initiation. We included hospitalized adults (≥18 years), who were labeled as allergic to ≥1 BLA upon admission, who then received during their stay ≥1 dose of BLA. For the purposes of this study, BLA included only penicillins (eg, amoxicillin, ampicillin), cephalosporins, or beta-lactam beta-lactamase-inhibitors combinations (BLBLI, eg, amoxicillin/clavulanic acid, piperacillin/tazobactam). Patients who were labeled as allergic to carbapenems and/or to monobactam, for which lower rates of cross-reactivity are attributed,^2^ were excluded. Of note, during the study’s period, there were only penicillin-based BLBLI prescribed at SMC (cephalosporin-based BLBLI were not yet available). If a patient was treated with the same BLA class during different hospitalizations, we included and captured only the first hospitalization. If a patient was treated with different classes of BLA during the same hospitalization, we included this patient as a single “study case,” and both exposures (to both agents) were captured. If a patient was treated with different BLA classes during different hospitalizations, each hospitalization was captured separately and analyzed as a separate “study case.” Data was extracted from all available medical records. The severity of adverse reactions was graded according to the World Allergy Organization anaphylaxis guidelines.^18^ The risk for cross-reactivity based on the BLA side chains (ie, between the BLA agent/s the patient is labeled as allergic to and the BLA agent/s eventually administered to the patient) was stratified to low risk versus high risk, according to an established classification.^19^ We collected data pertaining to patients’ demographics, medical history and comorbidities, clinical parameters related to the initial reaction to BLA that incited the allergy labeling, parameters pertaining to the current BLA exposure, and clinical outcomes. Out-of-hospital mortality data was retrieved from the Israeli Ministry of Interior records. To avoid confounding, morbidity outcomes were analyzed among survivors only.

The statistical analyses were executed with SPSS (IBM©; v. 29.0, NY, USA). The predictors to develop an allergic reaction following the BLA challenge and the association of developing an allergic reaction with each of the captured hospitalization’s outcomes were queried with logistic and Cox regressions, respectively.

## Results

During the study period, there were 14,088 hospitalizations of 9,670 adult patients at SMC. Of those, 3,571 patients (37%) had a BLA allergy label upon admission to the hospital. The study cohort consisted of 1,171 study cases (33% of 3,571), who were labeled as allergic to BLA but still did receive BLA during their hospitalization. There were 798 patients (68%) who were allergic to penicillins, 154 (13%) to penicillin-based BLBLI, 147 (12.5%) to cephalosporins, and 72 (6%) to various BLA combinations. The mean age of patients was 69.3 ± 19.4 years, and 785 (67% of 1,171) were women. Among nine hundred (900) patients (77%), details pertaining to the type of past allergic reaction were not provided by the patient nor caregivers. Among 1,072 patients (92%), the reason for the BLA challenge was not documented by staff.

Of the cohort of 1,171 patients, there were 30 patients (2.6%) who did develop an allergic reaction following challenge to BLA. All reactions were mild (ie, grade 1–2).^20^ Twelve of these patients (40%) did not receive any anti-allergic therapeutic management following the reaction (only BLA discontinuation and follow-up). The main manifestation of the reaction was an unspecified rash (n = 16, 53%), which resolved in less than a week in most patients (n = 18, 95%). The median time from BLA challenge to reaction was 1 day (IQR 0–5 days).

The univariate comparisons between the 30 patients who developed an allergic reaction and the 1,141 patients who did not develop an allergic reaction are depicted in Table 1. There were no demographic differences between groups. Significant statistical associations of parameters associated with the development of an allergic reaction following BLA challenges were (1) patients with documentation of the past allergic reaction (vs patients with no documentation, 50% vs 24%, *P* < .001), (2) patients with an atopic background (eg, asthma, atopic dermatitis, allergic rhinitis, food allergy, 20% vs 8%, *P* = .02), (3) patients challenged with the same type of BLA as labeled (vs those challenged with a different class of BLA, 33% vs 17%, *P* = .03), (4) high risk for cross-reactivity between the BLA side chains of the labeled and the challenged agent (5.6% vs 1.9%, *P* = .002),^19^ and (5) patients who received antihistamines at some point during hospitalization prior to the BLA-challenged event (10% vs 1%, *P* = .007).^16^

**Table 1.** Predictors and outcomes associated with allergic reaction following challenge to BLA among inpatients with BLA allergy labeling (Shamir Medical Center, 2019–2020)

Parameter			Developed an allergic reaction (n = 30)	Did not develop an allergic reaction (n = 1141)	Statistics	
			Number (%)^a^	Number (%)^a^	OR (95% CI)	*P* value
Demographics and atopic background	Age (years), mean ± SD		68 ± 13.8	69.4 ± 20		0.6
	Elderly (over 65 years old)		17 (56.7)	783 (68.6)	0.6 (0.3–1.2)	0.2
	Female gender		19 (63.3)	766 (67.1)	0.8 (0.4–1.8)	0.7
	Elixhauser Comorbidity Indexes^21^	Points, mean ± SD	7.8 ± 8.8	6 ± 7.3		0.3
		Percentage (ie., the probability of inpatient death), median (IQR)	76 (69–82)	78 (72–82)		0.34
	Atopic background (asthma, atopic dermatitis, allergic rhinitis, food allergy)		6 (20)	92 (8.1)	2.86	0.02
	Allergy to other medication except BLA		13 (43.3)	450 (39.4)	1.17 (0.6–2.4)	0.7
Parameters related to the initial allergic reaction	Class of BLA	Penicillin	19 (63)	779 (68)	0.8 (0.4–1.7)	0.6
		BLBLI	3 (10)	151 (13)	0.7 (0.2–2.4)	0.8
		Cephalosporins	5 (17)	142 (12)	1.5 (0.6–4.1)	0.4
		Combination of BLAs	3 (10)	69 (6)	1.7 (0.5–5.8)	0.4
	Year of BLA allergy initiation		2016 (2015–2016)	2016 (2006–2018)	0.9	
	Reason for allergy labeling as depicted in charts	Past allergic reaction	15 (50)	268 (24)	3.3 (1.6–6.8)	< .001
		Unknown/not documented	15 (50)	872 (76.4)	0.3 (0.1–0.6)	< .001
	Initial allergic reaction to the offending BLA	Immediate urticaria	0	8 (0.7)		> .99
		Non-immediate rash	12 (40)	175 (15.5)	2.7 (1.3–5.4)	0.004
		Angioedema	1 (3.3)	17 (1.6)	2.3 (0.3–18)	0.4
		Anaphylaxis	1 (3.3)	8 (0.7)	5 (0.6–40)	0.2
		Dyspnea	0	9 (0.8)		> .99
		Gastrointestinal	2 (6.6)	38 (3.4)	2.1 (0.5–9)	0.3
		SCAR	0	1 (0.1)		> .99
		Other	0	22 (1.9)		> .99
	Duration of rash	< 24 h	0	4 (22)		> .99
		1–7 d	1 (3.3)	12 (67)		> .99
		> 7 d	0	2 (11)		> .99
	Treatment	No treatment	0	4 (13.3)		> .99
		AH	1 (3.3)	13 (43.3)	1.3 (0.1–23)	> .99
		SCS	1 (3.3)	5 (16.7)	5 (0.3–94)	0.3
		Combination of both	0	7 (23.3)		> .99
Course of index hospitalization	Admission ward	Internal medicine	17 (57)	669 (59)	0.9 (0.4–1.9)	0.8
		Surgery	8 (27)	301 (27)	1.9 (0.8–4.6)	0.2
		Adult ICU	1 (3.3)	21 (2)	1.8 (0.2–14)	0.4
		OB/GYN	1 (3.3)	23 (2)	1.7 (0.2–13)	0.5
		ER	5 (17)	175 (12)	1.1 (0.4–3)	0.8
	SCS administration during hospitalization before BLA challenge		5 (16.7)	154 (13.5)	1.3 (0.5–3.4)	0.6
	AH administration during hospitalization before BLA challenge		3 (13)	13 (1.1)	9.6 (2.6–36)	0.007
Parameters related to the BLA challenge	Group of BLA received during hospitalization	Penicillin	0	20 (1.8)		> .99
		BLBLI	6 (20)	92 (8.1)	2.9 (1.1–7.2)	0.02
		Cephalosporin	20 (67)	972 (85)	0.5 (0.2–1)	0.06
		Combination of classes	4 (13)	57 (5)	3 (1–8.7)	0.07
	Matching BLA label to BLA received	PCN^c^-PCN^c^	3 (13)	42 (4.2)	7 (2–25)	0.01
		PCN^c^-CPN	16(54)	846 (74.1)	0.6 (0.2–1.3)	0.2
		CPN-PCN^c^	3 (13)	65 (6.4)		> .99
		CPN-CPN	1 (4.3)	67 (6.6)	0.6 (0.09–5)	> .99
	Perfect matching of the beta-lactam class^b^		6 (23)	121 (11)	2.3 (0.9–6)	0.07
	High risk for cross-reactivity based on the BLA agents side chains		12 (5.6%)	18 (1.9)	3.1 (1.5–6.5)	0.002
	Reason for choosing to administer BLA	Accidental	0	5 (0.5)		> .99
		Infectious Disease consultation	7 (25)	174 (15)	1.9 (0.8–4.4)	0.2
		Allergist consultation	0	2 (0.2)		> .99
		Attending physician discretion	6 (22)	288 (25)	0.8 (0.3–2)	0.6
		Unknown/non-documented reason	18 (64)	698 (86)	1.1 (0.5–2.5)	0.7
Outcomes	In-hospital death		5 (17)	128 (11)	1.6 (0.6–4.2)	0.4
	90-d mortality (following BLA administration)		7 (23)	219 (19)	1.3 (0.5-3)	0.6
	Among survivors of the index hospitalization only	Number of days from antibiotic administration to discharge or death	5 (3–13)	4 (2–8)		0.2
		Discharge to long-term care facility	5 (20)	92 (9.7)	2.3 (0.9–6.4)	0.1
		Functional status deterioration at discharge	8 (32)	198 (20)	2 (0.8–4.6)	0.1
		Additional hospitalizations in the 6 mo following the index hospitalization	8 (32)	379 (38)	0.8 (0.3–1.8)	0.6

Note. CI, confidence interval; IQR, interquartile range; OR, odds ratio; SD, standard deviation; BLBLI, beta-lactam beta-lactamase inhibitor combinations (only penicillin-based combinations were available at the time); BLA, beta-lactam antimicrobials (penicillin, cephalosporins, BLBLI); SCAR, severe cutaneous adverse reaction; AH, antihistamines; SCS, systemic corticosteroids; PCN, penicillin; CPN, cephalosporin; ICU, intensive care unit; OB/GYN, obstetrics and gynecology.

a Percent is presented as the “valid” percent, that is, after excluding the patients with missing information from the denominator.

b Perfect matching implies that the patient received the exact same BLA as in the allergy label.

c Penicillin includes also BLBLI.

In multivariate analysis, the independent predictors to develop an allergic reaction following BLA challenge were documented reactions in the past (aOR = 2.8; 95% CI,1.3–6), atopic background (aOR = 2.7; 95% CI, 1.1–6.8), high risk for cross-reactivity (based on the BLA side chains) between the labeled and the challenged agents (aOR = 2.9; 95% CI, 1.3–7.2),^19^ and antihistamines administration prior the BLA challenge event (aOR = 9.9; 95% CI, 2.6–38).

As depicted at the bottom of Table 1, both morbidity (after excluding the patients who died) and mortality outcomes were similar between groups. In separate multivariate outcome models, no independent associations with any worse outcomes were associated with the development of an allergic reaction following the BLA challenge.

## Discussion

In this retrospective investigation, we reviewed the charts of 14,088 hospitalized patients and found 1,171 inpatients who were labeled as allergic to BLA but still did receive a BLA during their admission. The most significant findings of this study are its descriptive statistics, that is, the fact that nearly 40% of hospitalized adults are labeled as allergic to BLA, and of those, 33% still do get BLA. The rate of BLA labeling was surprisingly higher than the rates previously reported from other centers, though most publications provided the specific rates associated with penicillin allergy, not the aggregated rates associated with all BLA agents.^1,9–11^ This, put together with the significant BLA challenging rates, which were not done as part of a designated stewardship plan, further highlights the burden imposed on patients and facilities, due to this common clinical scenario. Another finding is that 67% of the study’s cohort consisted of women. This is in concordance with other studies depicting higher allergy rates among women to drugs in general^22^ and to BLA specifically.^23,24^

Despite being perceived as a common phenomenon, only a minority of patients (n = 30, 2.6%) did develop an allergic reaction following the BLA challenge. Some of the “allergic reactions” developed up to 5 days following the BLA exposure, a finding that probably represents a benign delayed maculopapular rash, which is considered the most common reaction to BLA.^25^ This might further imply to the limited reliability of data pertaining to allergic manifestations, which are extracted retrospectively from charts). All reactions were mild, grade 1–2,^20^ and nearly half of the patients did not require any pharmaceutical intervention. This finding has been reported from other centers as well,^26–28^ further supporting proactive efforts to implement effective and safe BLA allergy de-labeling interventions. In a recent large study involving 2 large healthcare systems from 2 regions in California, USA, in which the default alert to avoid cephalosporines in cases of penicillin allergy was removed from the electronical medical record,^23^ the amount of cephalosporines given during admission dropped from 47% to 18%, while no more allergic reactions nor worse clinical outcomes were recorded.^16^

There were several independent predictors to develop a reaction following the BLA challenge. Reactions were more common among patients with documentation in their chart regarding their initial manifestations resulting in a BLA label, implying a higher probability of having some kind of reaction in the past. Atopic background of any sort was an additional independent predictor for an allergic reaction upon BLA challenge, as was antihistamines administration during the index hospitalization, prior to the BLA challenge event. This raises speculations regarding the accuracy of the data documentation, as extracted retrospectively from charts, because drug allergies are not necessarily related to atopic backgrounds.^29^ The 2 latter independent predictors for a reaction might suggest that some patients were mistakenly perceived to develop an allergic reaction due to symptoms such as pruritus or the use of antihistamines, due in part to their atopic background.^29^ An additional independent predictor was the risk for cross-reactivity between the labeled and the challenged agents, based on the BLA side chains.^19^ Because the most common challenge among patients labeled as allergic to penicillins was ceftriaxone (Table 1), this further supports the effectiveness and safety of the empiric intervention as implemented in the study from the Kaiser Permanente health systems in California (USA).^23^

Developing an allergic reaction following the BLA challenge was not associated statistically, with any worse clinical outcomes (Table 1). Some of the outcomes were worse among the 30 patients who developed a reaction (statistically insignificant), but these statistical trends might relate to the usage of non-BLA second-line antimicrobial agents,^3,13,14^ not to the allergic reactions per se, which were all very mild. In separate outcome analyses, none of the outcomes was independently associated with an allergic reaction following the BLA challenge.

This study has several inherent limitations, associated with its retrospective chart-review-based design, from a single institution. Important epidemiological information, particularly pertaining to the initial reaction that elicited a BLA allergy label, is not documented appropriately in medical charts. The reasoning for BLA challenges is also non-uniformly documented. Additionally, the prevalence of anaphylactic reactions to BLA is low, and therefore this study population was not powered to detect such an increment in risk. However, our findings are consistent with the current literature and emphasize that there is an exaggerated incidence of mistaken BLA allergy labels. Furthermore, the mild reactions that did develop in some cases do not justify the complete avoidance of the most important antimicrobials in our therapeutic armamentarium, particularly those considered to be of low risk for cross-reactivity (between the labeled and challenged agents), based on the BLA side chains.^19^

In summary, in this study, we found that nearly 40% of inpatient adults at SMC are flagged with a BLA allergy label, but still, 1 of every 3 patients receives a BLA, with an extremely high safety profile. This should prompt proactive interventions to remove the BLA allergy label from low-risk patients, to improve health and fiscal outcomes among individual patients and health institutions.
